# The Role of Preoperative Antibiotics in Osteosynthesis of the Hand and Wrist: A Retrospective Analysis

**DOI:** 10.3390/jcm14248877

**Published:** 2025-12-15

**Authors:** Anja Hunziker, Ilja Kaech, Brigitta Gahl, Konrad Mende, Dirk J. Schaefer, Alexandre Kaempfen

**Affiliations:** 1 Department of Plastic, Reconstructive, Aesthetic and Hand Surgery, University Hospital of Basel, 4031 Basel, Switzerland; anjasarah.hunziker@gmail.com (A.H.); ilja.kaech@gmail.com (I.K.); konradmende@sonnenhof.ch (K.M.); dirk.schaefer@usb.ch (D.J.S.); 2 Department of Plastic and Hand Surgery, Cantonal Hospital St. Gallen, 9007 St. Gallen, Switzerland; 3 Surgical Outcome Research Center, University Hospital Basel and University of Basel, 4031 Basel, Switzerland; brigitta.gahl@usb.ch; 4 Orthopedics Sonnenhof Bern, 3000 Bern, Switzerland

**Keywords:** hand surgery, osteosynthesis, antibiotic prophylaxis, preoperative antibiotics, postoperative infection, surgical site infection, infection prevention

## Abstract

**Background**: Preventing postoperative infections in hand surgery is an important factor for achieving sustainable results of surgical procedures. To prevent infections, especially when implants are used, preoperative prophylactic antibiotics are applied in adherence to predominantly national guidelines, which are not specifically tailored to hand surgery. However, several studies related to elective soft tissue hand surgery indicate that the preoperative use of antibiotics does not reduce the incidence of postoperative infections. Evidence regarding their efficacy in osteosynthesis of the hand and wrist remains limited. **Methods**: In this retrospective study, we analyzed 542 adult patients who underwent hand or wrist osteosynthesis between 2016 and 2019 at our university center. They were enrolled in an antibiotic treatment group and a control group without antibiotic treatment. The prophylaxis group (P) underwent surgery in the main operating theater under intravenous anesthesia, whereas the non-prophylaxis group (NP) was treated under WALANT (Wide Awake Local Anesthetic No Tourniquet) in an outpatient operating theater without receiving preoperative antibiotics. Theater construction and installation were otherwise similar, and both were classified as grade 1 theaters. We applied propensity modeling and inverse probability of treatment weighting (IPTW) to achieve balanced treatment groups with respect to risk factors for infection, and we calculated the odds ratio of prophylaxis and infection. Inclusion factors for risk of infection were age, female sex, smoking, diabetes, metabolic disease, inflammatory disease, substance abuse, cardiovascular disease, hepatopathy, renal disease, polytrauma, open fracture, being a manual worker, and occupational accidents. To assess the severity of the cases, we considered whether the fractures were intraarticular, multi-fragmentary, or open, and we collected data on the types of surgical implants that were used. **Results**: No significant association was found between antibiotic prophylaxis and postoperative infection rate (infection rate P: 3.86%; NP: 3.27%; unadjusted OR: 1.19; adjusted OR after IPTW: 1.09). In terms of risk factors, there was an insignificant trend of higher infection rates in the subgroups smoking, cardiovascular disease, open fracture, occupational accident, and open fixations. **Conclusions**: In this cohort, routine use of preoperative antibiotics in hand osteosynthesis did not reduce infection rates. The effectiveness of the widespread standardized application of prophylactic antibiotics to reduce the risk of postoperative infections in osteosynthesis of the hand and wrist remains debatable. Our findings set the basis for further prospective studies aiming at clearer guidelines for evidence-based perioperative patient care.

## 1. Introduction

Postoperative wound infections after hand surgery are uncommon, but when they occur, they are a major source of morbidity, potentially leading to loss of function due to stiffness, scarring, or even amputation of the involved parts [1]. The postoperative infection rate is reported to be less than 1.5% after elective soft-tissue hand operations and below 4% after hand surgery in general [1,2]. In an effort to prevent postoperative wound infections, perioperative antibiotic prophylaxis is commonly utilized. However, several studies related to elective soft tissue hand surgery indicate that the preoperative use of antibiotics does not significantly decrease infection rates [1,3,4]. There is also evidence suggesting that antibiotic administration does not offer statistically significant advantages over placebo in dirty machinery-caused hand injuries involving joints and bones [2].

Whereas most studies focus on soft tissue hand surgery, preoperative antibiotics in osteosynthesis of the hand have received limited attention, and the available evidence remains scarce.

Due to the low infection rates in hand surgery in general, most existing studies are statistically underpowered [1]. This leads to a scarcity of specific guidelines. Still, one must consider the possibility of adverse events related to antibiotic use, such as potentially lethal Clostridium difficile colitis and allergic reactions [3]. In the absence of specific guidelines tailored to hand surgery, guidelines for large bones and joints are often used, resulting in a high potential for antibiotic resistance.

Therefore, the issue of preoperative administration of antibiotics in osteosynthesis of the hand and wrist requires further attention. In the Hand and Plastic Surgery Department at the University Hospital of Basel, we perform hand surgery, including osteosynthesis, in either the main operating theater or in the ambulatory operating theater. Both operating theaters are equipped with laminar flow ventilation, have identical surgical standards, and are used by surgeons of all levels. However, in the ambulatory operating theater, which is used for local anesthesia procedures only, the need for an IV line seems cumbersome, and the administration of oral antibiotics would have increased procedural time, thereby slowing efficiency in turnover time. Therefore, no antibiotics are administered. In contrast, such prophylaxis is typically applied in our main operating room by anesthetists, who always install an IV line for anesthesia safety. To critically assess the safety aspect regarding postoperative infection rates of not administering antibiotics, we conducted a retrospective analysis. Since the evidence against perioperative antibiotic administration for soft tissue procedures seemed clear, our study focuses on implants. The aim of this study was to evaluate whether preoperative antibiotic prophylaxis reduces postoperative infection rates after osteosynthesis of the hand or wrist. We hypothesized that it does not significantly lower the risk of postoperative infections compared with no preoperative prophylaxis.

## 2. Materials and Methods

We conducted a retrospective cohort study including patients over 18 years of age who underwent bone fixation with implants on the hand or wrist between 2016 and 2019 at our university hospital, regardless of the underlying pathology. Open fractures were deliberately included because the aim was to evaluate all implant-related procedures in routine clinical practice, including higher-risk cases. Excluding them would have reduced the generalizability of the findings and introduced selection bias. Patients undergoing implant removal and those with multiple surgeries were excluded to prevent indication bias and statistical distortion. Implant removal cases represented removal-only procedures. We excluded implant removal cases because these procedures are minimally invasive, substantially shorter, and clinically not comparable to primary fixation. Their inclusion would have biased operative characteristics and confounded infection risk estimation. If a patient underwent repeated surgeries during the study period, only the first eligible procedure was included to avoid correlated observations. Data were obtained from electronic health care records. The dataset was anonymized to ensure patient confidentiality. The study was approved by the Ethics Committee of Northwestern and Central Switzerland (EKNZ) and informed consent was obtained from all subjects involved in the study. Data abstraction was performed by a single reviewer.

We defined either presence or absence of postoperative infection as primary outcome and divided patients into a prophylaxis (P) and non-prophylaxis (NP) group. The allocation to the respective operating theater (ambulatory WALANT setting vs. main operating room) was the primary determinant of whether patients received preoperative antibiotic prophylaxis. All signs of infection were retrieved from case files. As infections were not consistently classified, any description of clinical signs of an infection (swelling, redness, pus, and increased local pain), as well as treatment with antibiotics and wound debridement were considered an infection. Microbiological confirmation was not consistently available and therefore not used as a criterion. We intentionally applied this broad definition to minimize missed infections due to inconsistent documentation in retrospective records. This approach reduces the risk of underreported cases but limits comparability with studies using stricter surgical site infection (SSI) criteria.

To enable a more detailed subgroup analysis, we evaluated various potential risk factors. In addition to age and sex, the factors smoking, diabetes, other metabolic diseases, inflammatory diseases, substance abuse, cardiovascular diseases, obesity, hepatopathy, renal insufficiency, occupational accident, manual worker, and polytrauma were noted. Each factor was coded as binary (yes/no). Comorbidities were identified through ICD-coded diagnoses and physician chart documentation. Incomplete records were excluded if essential variables for propensity modeling or outcome assessment were missing. To further assess the complexity of the surgery and the fracture, we also collected data on the type of surgical implant used (Kirschner wire, screw, plate, cannulated compression screws (CCS), fixateur externe), and the complexity of the fracture pattern (intraarticular, open fracture, open fixation surgery, or multi-fragmentary). Open fixation refers to procedures requiring an open surgical approach, as opposed to percutaneous or minimally invasive fixation.

For statistical analysis, we applied propensity modeling to achieve balanced treatment groups with respect to the risk factors for infection mentioned above. Propensity scores were estimated using logistic regression based on all predefined covariates (age, sex, smoking, diabetes, metabolic and inflammatory diseases, substance abuse, cardiovascular diseases, obesity, hepatopathy, renal insufficiency, occupational accident, manual labor, polytrauma, and fracture complexity variables).

Propensity scores estimate the likelihood of receiving prophylaxis based on covariates. The *p*-values and the respective standardized differences (SD) give insight into the balance of risk factors. Positive values show higher means in the NP, whereas negative values indicate higher means in the P group. Values close to zero (SD < 0.1) demonstrate balance. Low *p*-values (*p* < 0.05) suggest significant differences in the balance of the risk factors between the groups.

We derived normalized inverse probability of treatment weighting (IPTW) by truncating all weights at 10. IPTW creates a synthetic sample using propensity scores and balances the distribution of covariates. We calculated standardized differences between treatment groups before and after IPTW to check whether balance was achieved, independent of statistical power. We calculated an odds ratio (OR) with a 95% confidence interval (CI) of prophylaxis and infection using logistic regression after IPTW. A two-sided significance level of α = 0.05 was used. Continuous covariates were kept continuous in the regression models, and categorical variables were treated as binary factors. Model fit and multicollinearity were assessed descriptively. Two sensitivity analyses were performed: first, a crude OR; second, a model adjusted for age, manual labor, and cardiovascular disease. Information on surgical implants used was not included into propensity score calculation which must be based on pre-treatment variables.

Continuous variables were presented as mean ± standard deviation if normally distributed, or as geometric mean with standard deviations back-transformed from the log scale if distribution was skewed. Categorical variables are shown as numbers with percentages and compared using logistic regression.

Finally, we analyzed the infection group separately, comparing risk factor prevalence with the overall population. We also examined infection rates within subgroups and assessed whether risk factors were more common among patients with infection.

All analyses were carried out using Stata 16.0 (StataCorp LLC, College Station, TX, USA) and Microsoft Excel for Microsoft 365, Version 2510 (Build 16.0.19328.20178) (Microsoft Corporation, Redmond, WA, USA).

## 3. Results

A total of 542 patients met the inclusion criteria. Of these, 389 (71.8%) received preoperative antibiotics (prophylaxis group, P) during surgery in the main operating theater, while 153 (28.2%) underwent surgery without antibiotic prophylaxis (non-prophylaxis group, NP) in the ambulatory operating room. The distribution of patients across both groups reflects the established institutional workflow, where IV access is routinely established in the main operating theater but not in the ambulatory operating room.

As shown in Table 1, the overall infection rate of the entire study population was 3.69%, with a total of 20 infections. There were 15 infections in the P group, corresponding to a 3.86% infection rate. In the NP, there were 5 infections, corresponding to an infection rate of 3.27%. Table 2, Table 3, Table 4, Table 5 and Table 6 summarize the results of the more profound analysis of the different risk factors and the infection rates in these various subgroups. The infection rate was higher in smokers (4.17%), cardiovascular disease (5.36%), open fracture (7.69%), occupational accident (12.12%), and open fixation (3.80%). For these risk factors, in both the antibiotic and non-antibiotic groups, infection rates increased, showing a generally comparable trend. In patients of the subgroups smoking (P: 3.97%; NP: 4.88%) and occupational accidents (P: 11.90%; NP: 12.50%), the infection rate was slightly higher in the NP group. The opposite was observed in the subgroups open fracture (P: 7.84%; NP: 7.14%) and open fixation (P: 4.01%; NP: 2.90%), with a higher infection rate in the P group. The subgroup cardiovascular disease showed a more noticeable difference in infection rates (P: 4.44%; NP: 9.09%) between the antibiotic and non-antibiotic groups. Because the absolute number of infections in several subgroups was low, these comparisons should be interpreted with caution. Diabetes mellitus, metabolic diseases, substance abuse, inflammatory diseases, obesity, hepatopathy, renal insufficiency, and polytrauma are not listed in the tables because of the much lower number of patients, which did not allow any analysis of the data.

Of the overall patient population, 35.4% were smokers, 20.7% had cardiovascular disease, 12% had an open fracture, 12.2% had an occupational accident, and 68% had an open fixation surgery. In the infection group, 40% were smokers, 30% had a cardiovascular disease, 25% had an open fracture, 40% had an occupational accident, and 70% had an open fixation surgery. This reflects that infections occurred more frequently in patients with pre-existing comorbidities or more complex injury patterns.

Table 7(a) summarizes patients’ characteristics and risk factor distributions in both groups to identify potential imbalances. All risk factors except diabetes mellitus (NP: 4.6%; P: 4.1%), being a manual worker (NP: 30.1%; P: 28.8%), and occupational accidents (NP: 15.7%; P: 10.8%) were more prevalent in patients who had received preoperative antibiotics. Although being more prevalent in one group, diabetes mellitus (*p* = 0.810; SD = 0.023) and being a manual worker (*p* = 0.769; SD = 0.028) are well distributed, whereas occupational accidents had a *p*-value higher than 0.05 and a standardized difference higher than 0.1 (*p* = 0.119; SD = 0.145). In summary, only occupational accidents were notably more prevalent in the NP. The risk factors with the greatest disparity (*p* < 0.05) were age (*p* = 0.002; SD = 0.3), smoking (*p* = 0.009; SD = −0.258), cardiovascular disease (*p* = 0.025; SD = −0.226), obesity (*p* = 0.001; SD = −0.482), polytrauma (*p* = 0.046; SD = −0.224), open surgery (*p* = 0.000; SD = −0.730), and intraarticular (*p* = 0.000; SD = −0.461). More evenly distributed factors with a slightly higher percentage observed in the P group are female sex (*p* = 0.174; SD = −0.133), metabolic disease (*p* = 0.357; SD = −0.091), inflammatory disease (*p* = 0.217; SD = −0.127), substance abuse (*p* = 0.082; SD = −0.178), hepatopathy (*p* = 0.174; SD = −0.149), renal insufficiency (*p* = 0.084; SD = −0.197), open fracture (*p* = 0.179; SD = −0.134), and multi-fragmentary (*p* = 0.092; SD = −0.164). Due to the low percentage of patients: obesity (NP: 1.3%; P: 13.6%), hepatopathy (NP: 1.3%; P: 3.6%), renal insufficiency (NP: 1.3%; P: 4.6%), and polytrauma (NP: 2%; P: 6.4%) have to be looked at more carefully. These imbalances underline the need for weighted statistical approaches.

**Figure 1:**
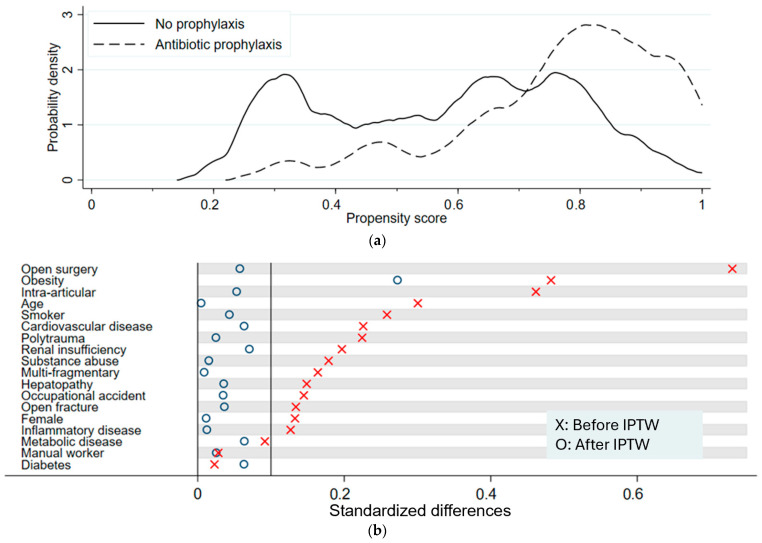
(**a**)**.** Distribution of propensity scores before IPTW. The solid line represents the non-prophylaxis (NP) group, and the dashed line represents the prophylaxis (P) group. The curves illustrate the degree of overlap and imbalance in pre-treatment covariates before weighting. (**b**)**.** Standardized differences before and after IPTW. Crosses represent standardized differences before weighting, and circles represent standardized differences after weighting. The plot demonstrates that IPTW substantially reduced standardized differences across covariates, indicating improved balance between groups.

Figure 1a illustrates the distribution of propensity scores across the two groups. With the probability density on the y-axis, the graph illustrates the distribution of propensity scores across the NP and the P group. Overlapping graphs suggest balance between groups. The P group has a peak at propensity scores around 0.8, whereas the probability density of the NP is more evenly distributed, with three peaks at propensity scores around 0.3 and between 0.6 and 0.8. The divergence between the curves representing the two main groups indicates a certain imbalance in the probability of receiving antibiotics. These results show that certain subgroups are more prevalent in the P group and, therefore, were more likely to receive preoperative antibiotics. This imbalance reflects the underlying clinical decision-making, where patients with more complex injuries tended to receive prophylaxis.

The results after applying IPTW are shown in Table 7(b) *p*-values were larger (*p* > 0.05, range of 0.6–0.9), suggesting a well-balanced distribution of risk factors. Only obesity stood out with a lower *p*-value of 0.115. Figure 1b shows a visualization of standardized differences before and after applying IPTW. Standardized differences after IPTW dropped to below 0.1, indicating no meaningful difference in all variables except for obesity, which had a value of 0.273. Thus, IPTW substantially improved comparability between groups.

**Figure 2:**
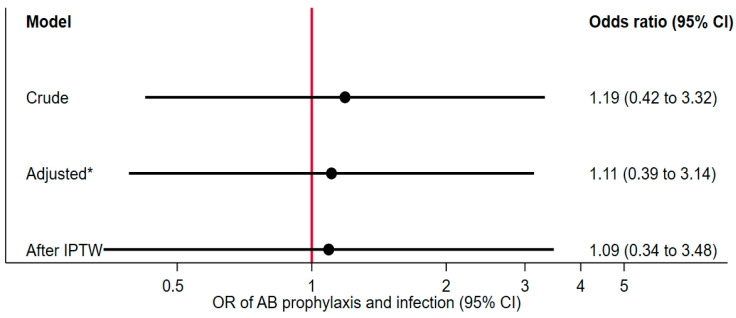
Odds ratio, * for age, manual worker, and cardiovascular disease.

Figure 2 shows the odds ratio calculation with the associated 95% confidence interval (CI) before and after IPTW and after adjustment for age, being a manual worker, and cardiovascular disease. We did not find an association between the absence of preoperative antibiotic treatment and the postoperative infection rate. The crude OR was 1.19 (CI: 0.49 to 3.32), indicating that infection frequency was slightly higher in patients who had received preoperative antibiotic prophylaxis. Neither adjustment (OR = 1.11, CI: 0.39 to 3.14) nor IPTW (OR = 1.09, CI: 0.34 to 3.48) substantially altered the odds ratio. All confidence intervals crossed 1.0, indicating the absence of a statistically significant association.

Table 8 presents the surgical details of which implants were used and their distribution in the prophylaxis and the non-prophylaxis group. In the non-prophylaxis group (NP) with 153 patients, the most frequently used implants were Kirschner wires, utilized in 81 patients (53%). Other implants used in the surgery were CCS in 32 patients (21%), plates in 25 patients (16%), and other screws in 19 patients (12%). We also listed fixateur externe and other implants, both of which were used in 1 patient (0.65%). In the prophylaxis group (P), comprising 391 patients, Kirschner wires were also prominently used in 153 patients (39%). This was followed by plates which were used in 115 patients (30%), screws in 87 patients (22%), and CCS in 76 patients (20%). Fixateur externe was utilized in 5 patients (1.3%), and other implants were listed in 7 patients (1.8%) of the prophylaxis group. We used logistic regression to compare implant distribution in the two groups. Kirschner wires (*p* = 0.005), screws (*p* = 0.008), and plates (*p* = 0.002) showed *p*-values lower than 0.05, indicating a certain difference in the surgical implants between the two groups. CCS (*p* = 0.72) and fixateur externe (*p* = 1.00) were balanced with larger *p*-values. These differences likely reflect variation in case complexity between the two operating environments.

## 4. Discussion

In this retrospective cohort of 542 adult patients undergoing osteosynthesis of the hand or wrist at a tertiary university hospital, no statistically significant association was found between abstaining from prophylactic preoperative antibiotics and postoperative infection rates. After adjustment and IPTW, the odds ratio remained close to 1 (crude OR 1.19, 95% CI 0.42–3.32; adjusted OR 1.11, 95% CI 0.39–3.14; IPTW OR 1.09, 95% CI 0.34–3.48), indicating no measurable protective effect of prophylactic antibiotics in our cohort. Interestingly, even in high-risk subgroups, such as open fractures and occupational accidents, there was no clear benefit of preoperative prophylactic antibiotics. For example, the infection rate in the subgroup occupational accidents remained similar after preoperative antibiotics (P: 11.9%; NP: 12.5%). The difference was minimal, rendering it uncertain whether preoperative antibiotics provided a real benefit.

The infection rate observed in our cohort (overall 3.69%) appears elevated compared to typical SSI rates in hand surgery, which are generally reported as <1% in clean cases and <5% overall. This discrepancy likely reflects the wider and pragmatic infection definition used in our study. Rather than relying solely on clear documentation of SSI or microbiological confirmation, we also included signs of infection to reduce bias due to missing documentation. Wormald et al. found that SSI in hand surgery in retrospective studies are usually more difficult to identify due to poorer medical record documentation [5]. Also, minor infections and complications are missing when focusing only on clearly documented SSI [6]. However, this method limits the differentiation between superficial, deep and implant-related infections.

Our findings are consistent with previous studies. The current consensus is that preoperative antibiotics are not supported in elective soft tissue hand surgery [1,7]. Only a small number of studies specifically address antibiotics in hand surgery involving hardware and bones [8]. In our study evaluating osteosynthesis in the hand, we therefore excluded soft tissue hand surgery. Our findings align with those of Feldman et al., who similarly found no benefit of prophylactic antibiotics in closed hand fractures [9]. Furthermore, these results are consistent with the findings of Aydin et al. and Metcalfe et al., who found no statistically significant effect of preoperative antibiotics in contaminated or open hand injuries [2,10]. They concluded that the hand, due to its better blood supply supporting the healing process, could be considered an immunologically privileged area, leading to a decrease in infection rate and severity. In case of an infection, early effective debridement and irrigation are sufficient treatments [10].

We found that patients receiving preoperative antibiotics tended to have more comorbidities and risk factors. This may suggest that in the absence of clear guidelines, it is the individual surgeon deciding whether to administer antibiotics. Even after applying inverse probability of treatment weighting (IPTW) to balance baseline characteristics, the odds ratio for infection remained slightly above 1. This suggests residual confounding due to unmeasured factors such as fracture severity, contamination level, or duration of surgery. These factors could not be included in our dataset due to inconsistent documentation. Furthermore, a selection bias may have occurred regarding the allocation of patients to the main operating theater versus the outpatient operating room. Although the infrastructural differences between the theaters are minimal, surgeons in the central theaters might have benefited from better staffing and overall patient care. This could have had a positive effect on infection outcomes by reducing the surgeons’ workload and enhancing precision. On the other hand, more complex fractures may have been treated in the central operating theaters due to the availability of additional staff. We attempted to minimize this bias by including fracture patterns and comorbidities in the IPTW analysis. Further limitations exist, as prophylactic antibiotics may still have been administered postoperatively, because this study specifically focuses on preoperative intravenous antibiotic prophylaxis. Our broader infection definition was chosen to reduce the risk of missing relevant infections but likely led to a higher observed infection rate. This improves sensitivity but limits comparability with studies using stricter SSI definitions. Furthermore, the wide 95% confidence intervals, especially after IPTW (0.34 to 3.48), reflect the low event rate and suggest that our study may be underpowered to detect a small but potentially clinically relevant difference in infection rates between the two groups. This is a common limitation in hand surgery studies due to generally low infection rates.

Due to the limitations of the dataset and study design, these results cannot be interpreted as a definitive conclusion against the use of preoperative antibiotic prophylaxis. Instead, they highlight the challenges of retrospective research in hand surgery and underline the need for standardized definitions, consistent documentation, and institutional guidelines.

Future research should focus on prospective data collection with standardized SSI definitions and complete documentation of relevant clinical variables. Studies specifically addressing high-risk subgroups such as open fractures or heavily contaminated injuries are needed. Ideally, randomized controlled trials or well-designed prospective cohort studies could clarify whether selected patient groups benefit from preoperative antibiotic prophylaxis.

## 5. Conclusions

Our retrospective study shows no higher infection rate in the outpatient operating room, despite the general absence of preoperative antibiotics for osteosynthesis in the hand in this setting. Inverse probability of treatment weighting (IPTW) could largely weigh risk factors for patients receiving antibiotics preoperatively within our retrospective data. No specific patient subgroup was found to clearly benefit from preoperative antibiotic prophylaxis. These results, which align with other studies, provide a foundation for further investigations on this topic, aiming to create significant evidence to support clinical guidelines and safer perioperative patient care in hand surgery. Also, our study creates a basis for ethically justifying a prospective trial.

## Figures and Tables

**Table 1:** Overall infection rates in the study population.

Study Population	Total (n)	No Infection (n)	Infection (n)	Infection Rate (%)
Total	542	522	20	3.69%
Patients with AB	389	374	15	3.86%
Patients without AB	153	148	5	3.27%

**Table 2:** Infection rates in the subgroup smoking.

Subgroup Smoking	Total (n)	No Infection (n)	Infection (n)	Infection Rate (%)
Total	192	184	8	4.17%
Patients with AB	151	145	6	3.97%
Patients without AB	41	39	2	4.88%

**Table 3:** Infection rates in the subgroup cardiovascular disease.

Subgroup Cardiovascular Disease	Total (n)	No Infection (n)	Infection (n)	Infection Rate (%)
Total	112	106	6	5.36%
Patients with AB	90	86	4	4.44%
Patients without AB	22	20	2	9.09%

**Table 4:** Infection rates in the subgroup open fracture.

Subgroup Open Fracture	Total (n)	No Infection (n)	Infection (n)	Infection Rate (%)
Total	65	60	5	7.69%
Patients with AB	51	47	4	7.84%
Patients without AB	14	13	1	7.14%

**Table 5:** Infection rates in the subgroup occupational accident.

Subgroup Occupational Accident	Total (n)	No Infection (n)	Infection (n)	Infection Rate (%)
Total	66	58	8	12.12%
Patients with AB	42	37	5	11.90%
Patients without AB	24	21	3	12.50%

**Table 6:** Infection rates in the subgroup open fixation.

Subgroup Open Fixation *	Total (n)	No Infection (n)	Infection (n)	Infection Rate (%)
Total	368	354	14	3.80%
Patients with AB	299	287	12	4.01%
Patients without AB	69	67	2	2.90%

* open fixation = open surgical approach.

**Table 7:** (**a**). Patient characteristics before IPTW. (**b**). Patient characteristics after IPTW.

(**a**)				
**Risk Factors**	**No Prophylaxis,** **n = 153**	**Antibiotic Prophylaxis,** **n = 389**	**SD**	***p*** **-Value**
Age	39.2 ± 17.0	44.6 ± 19.0	0.300	0.002
Female	33 (21.6%)	106 (27.2%)	−0.133	0.174
Diabetes	7 (4.6%)	16 (4.1%)	0.023	0.810
Smoker	41 (26.8%)	151 (38.8%)	−0.258	0.009
Metabolic disease	8 (5.2%)	29 (7.5%)	−0.091	0.357
Inflammatory disease	5 (3.3%)	23 (5.9%)	−0.127	0.217
Substance abuse	9 (5.9%)	42 (10.8%)	−0.178	0.082
Cardiovascular disease	22 (14.4%)	90 (23.1%)	−0.226	0.025
Obesity	2 (1.3%)	53 (13.6%)	−0.482	0.001
Hepatopathy	2 (1.3%)	14 (3.6%)	−0.149	0.174
Renal insufficiency	2 (1.3%)	18 (4.6%)	−0.197	0.084
Polytrauma	3 (2.0%)	25 (6.4%)	−0.224	0.046
Open fracture	14 (9.2%)	52 (13.4%)	−0.134	0.179
Manual worker	46 (30.1%)	112 (28.8%)	0.028	0.769
Occupational accident	24 (15.7%)	42 (10.8%)	0.145	0.119
Open surgery	69 (45.1%)	305 (78.4%)	−0.730	0.000
Intra-articular	64 (41.8%)	250 (64.3%)	−0.461	0.000
Multi-fragmentary	46 (30.1%)	147 (37.8%)	−0.164	0.092
(**b**)				
**Risk Factors**	**No Prophylaxis,** **n = 153**	**Antibiotic Prophylaxis,** **n = 389**	**SD**	***p*** **-Value**
Age	43.6 ± 20.6	43.5 ± 19.8	−0.005	0.962
Female	38 (25.1%)	100 (25.6%)	−0.011	0.919
Diabetes	4 (2.8%)	15 (4.0%)	−0.063	0.464
Smoker	51 (33.6%)	139 (35.7%)	−0.043	0.709
Metabolic disease	8 (5.2%)	26 (6.7%)	−0.064	0.549
Inflammatory disease	8 (5.0%)	21 (5.3%)	−0.012	0.921
Substance abuse	14 (9.4%)	38 (9.8%)	−0.015	0.904
Cardiovascular disease	28 (18.3%)	81 (20.8%)	−0.063	0.597
Obesity	5 (3.4%)	40 (10.2%)	−0.273	0.115
Hepatopathy	4 (2.3%)	11 (2.9%)	−0.035	0.810
Renal insufficiency	4 (2.5%)	14 (3.7%)	−0.070	0.640
Polytrauma	7 (4.7%)	20 (5.3%)	−0.025	0.864
Open fracture	17 (10.9%)	47 (12.0%)	−0.036	0.747
Manual worker	41 (26.9%)	109 (28.0%)	−0.025	0.815
Occupational accident	19 (12.5%)	44 (11.3%)	0.035	0.735
Open surgery	102 (66.9%)	271 (69.6%)	−0.057	0.574
Intra-articular	85 (55.8%)	227 (58.4%)	−0.053	0.625
Multi-fragmentary	55 (35.8%)	141 (36.2%)	−0.009	0.938

**Table 8:** Implanted devices.

Surgical Implants	Total, n = 542	No Prophylaxis, n = 153	Antibiotic Prophylaxis, n = 389	*p*
Kirschner wire	234 (43%)	81 (53%)	153 (39%)	0.005
Screw	106 (20%)	19 (12%)	87 (22%)	0.008
CCS	108 (20%)	32 (21%)	76 (20%)	0.72
Plate	140 (26%)	25 (16%)	115 (30%)	0.002
Fixateur externe	6 (1.1%)	1 (0.65%)	5 (1.3%)	1.00
Other implants	8 (1.5%)	1 (0.65%)	7 (1.8%)	0.45

## Data Availability

The data that support the findings of this study are not publicly available due to ethical and privacy restrictions concerning patient confidentiality. Access to the data may be granted by the corresponding author upon reasonable request and with permission from the Ethics Committee of Northwestern and Central Switzerland (EKNZ), ensuring compliance with all applicable data protection regulations.

## Abbreviations

The following abbreviations are used in this manuscript:

CCS	Cannulated Compression Screws
CI	Confidence Interval
IPTW	Inverse Probability of Treatment Weighting
NP	Non-prophylaxis group (patients not receiving preoperative antibiotics)
OR	Odds Ratio
P	Prophylaxis group (patients receiving preoperative antibiotics)
SD	Standardized Difference
SSI	Surgical Site Infection
WALANT	Wide Awake Local Anesthetic No Tourniquet

## References

[1] Shapiro L.M., Zhuang T., Li K., Kamal R.N. (2019). The Use of Preoperative Antibiotics in Elective Soft-Tissue Procedures in the Hand: A Critical Analysis Review. JBJS Rev..

[2] Aydin N., Uraloǧlu M., Burhanoǧlu A.D.Y., Sensöz Ö. (2010). A Prospective Trial on the Use of Antibiotics in Hand Surgery. Plast. Reconstr. Surg..

[3] Bykowski M.R., Sivak W.N., Cray J., Buterbaugh G., Imbriglia J.E., Lee W.P.A. (2011). Assessing the Impact of Antibiotic Prophylaxis in Outpatient Elective Hand Surgery: A Single-Center, Retrospective Review of 8,850 Cases. J. Hand Surg..

[4] Li K., Sambare T.D., Jiang S.Y., Shearer E.J., Douglass N.P., Kamal R.N. (2018). Effectiveness of Preoperative Antibiotics in Preventing Surgical Site Infection after Common Soft Tissue Procedures of the Hand. Clin. Orthop. Relat. Res..

[5] Wormald J.C., Baldwin A.J., Nadama H., Shaw A., Wade R.G., Prieto-Alhambra D., Cook J.A., Rodrigues J.N., Costa M.L. (2023). Surgical Site Infection Following Surgery for Hand Trauma: A Systematic Review and Meta-Analysis. J. Hand Surg. Eur. Vol..

[6] Kistler J.M., Munn M., McEntee R., Ilyas A.M. (2023). Antibiotic Prophylaxis in Clean Hand Surgery: A Prospective Cohort Analysis of Major and Minor Complications. J. Hand Surg. Glob. Online.

[7] Mehta S., Court T., Graf A., Best C., Havlik R. (2023). The Impact of Clinical Practice Guidelines on Preoperative Antibiotic Administration for Carpal Tunnel Release. Hand.

[8] Rizvi M., Bille B., Holtom P., Schnall S.B. (2008). The Role of Prophylactic Antibiotics in Elective Hand Surgery. J. Hand Surg..

[9] Feldman G., Orbach H., Rozen N., Rubin G. (2021). Usefulness of Prophylactic Antibiotics in Preventing Infection after Internal Fixation of Closed Hand Fractures. Hand Surg. Rehabil..

[10] Metcalfe D., Aquilina A.L., Hedley H.M. (2016). Prophylactic Antibiotics in Open Distal Phalanx Fractures: Systematic Review and Meta-Analysis. J. Hand Surg. Eur. Vol..

